# Dodecylamine-promoted persistence of ultrasonication-generated microbubbles and its implications for quartz flotation

**DOI:** 10.1016/j.ultsonch.2026.108138

**Published:** 2026-10-26

**Authors:** Ming Xu, Haijun Zhang, Martin Rudolph

**Affiliations:** a Helmholtz-Zentrum Dresden-Rossendorf, Helmholtz Institute Freiberg for Resource Technology, Freiberg 09599, Germany; b School of Chemical Engineering and Technology, China University of Mining & Technology, Xuzhou, Jiangsu 221116, China

**Keywords:** Surfactant, Flotation, Microbubble, Ultrasonication, Gas–liquid interfacial tension

## Abstract

Dodecylamine (DDA), a cationic surfactant widely used in quartz flotation, can promote microbubble formation during ultrasonication; however, these ultrasonication-generated (*UG*) microbubbles may rapidly dissolve once the ultrasonic field is removed. This study hypothesizes that combining DDA with air oversaturation can markedly suppress post-ultrasonication microbubble dissolution and thereby enhance quartz flotation. *UG* bulk and surface microbubbles were characterized using dissolved-air measurements and optical imaging, while surface tension and contact angle measurements were used to elucidate the role of DDA. Flotation performance was evaluated using bubble pick-up and micro-flotation tests. DDA markedly increases the population of persistent *UG* microbubbles under air-oversaturated conditions, whereas no clear DDA-induced increase is observed under non-oversaturated conditions. This behavior is consistent with the coupled effects of air oversaturation and the DDA-induced reduction in gas–liquid interfacial tension, which together enhance the diffusive stability of microbubbles. The unexpectedly rapid decrease in bubble contact angle following bubble-solid contact in DDA solution suggests possible interfacial redistribution of DDA from the solid surface to the gas–liquid interface, which may accelerate the local reduction in gas–liquid interfacial tension. The resulting larger population of persistent microbubbles markedly enhances quartz-particle attachment to carrier bubbles and improves flotation recovery, highlighting the coupled roles of air oversaturation and surfactant adsorption in ultrasound-assisted flotation.

## 1 Introduction

Under ultrasonic irradiation, rectified diffusion arising from the repeated expansion and contraction of gas nuclei can promote the activation and growth of pre-existing nuclei even in gas-saturated liquids [1], [2], [3]. For a given nucleus and solution condition, rectified growth occurs only when the acoustic pressure amplitude exceeds a threshold value, which generally decreases with increasing bubble radius and decreasing gas–liquid interfacial tension [3], [4]. Therefore, surfactant adsorption at the gas–liquid interface, by lowering the interfacial tension, may facilitate the activation and growth of smaller gas nuclei and thereby increase the population of bubbles undergoing rectified diffusion [5], [6]. Surfactants can also increase the rate of rectified-diffusion-driven bubble growth [5], [6], [7]. Under strong acoustic oscillations, growing bubbles may become unstable and fragment into smaller daughter bubbles and gas nuclei that can seed further bubble growth. A reduction in interfacial tension promotes this process [8], [9]. Moreover, Lee and Kim [10] showed that increasing the concentration of sodium dodecyl sulfate (SDS) markedly increased the number of ultrasonication-generated (*UG*) bulk nanobubbles, partly by suppressing bubble coalescence and stabilizing small bubbles. Collectively, these studies indicate that surfactants can increase the microbubble populations during ultrasonication by influencing bubble activation, growth, fragmentation, coalescence, and interfacial stabilization [5], [6], [7], [8], [9], [10].

However, considerably less attention has been paid to the fate of *UG* microbubbles after the ultrasonic field is removed. The persistence of *UG* microbubbles after ultrasonication is particularly important for their subsequent application, given the growing use of stable micro- and nanobubbles in various engineering processes, such as water and wastewater treatment [11]. However, owing to their small size and the correspondingly high Laplace pressure, these bubbles are inherently susceptible to rapid dissolution. Our previous study [12] showed that gas oversaturation is essential for counteracting Laplace-pressure-driven dissolution and maintaining *UG* microbubbles after ultrasonication. A surfactant-induced reduction in surface tension can likewise lower the Laplace pressure and weaken the driving force for bubble dissolution. Therefore, the coupled effects of gas oversaturation and surfactant adsorption on the post-ultrasonication persistence and stability of *UG* microbubbles remain to be clarified.

This issue is particularly relevant to froth flotation, a key separation process in mineral processing and solid-waste valorization that exploits differences in particle wettability [13]. Ultrasonication pretreatment has been reported to enhance flotation through several mechanisms, including cleaning mineral surfaces [14], [15], creating additional active sites [16], promoting agglomeration [17], [18], and generating microbubbles [19]. During ultrasonic pretreatment, *UG* bulk microbubbles can attach to particle surfaces, forming surface microbubbles. Yang et al. [20] showed that *UG* surface microbubbles preferentially form on hydrophobic coal surfaces rather than on hydrophilic quartz surfaces. Kruszelnicki et al. [19] reported that ultrasonication pretreatment enhanced the flotation of copper-bearing shale in solutions containing 4-methyl-2-pentanol (MIBC) and potassium ethyl xanthate (KEX). Moreover, they identified *UG* nano/microbubbles as a major contributor to this enhancement. Previous studies have shown that surface microbubbles formed by gas nucleation driven by air oversaturation can enhance bubble-particle interactions [21], [22], [23]. These findings indicate that, for *UG* microbubbles to contribute effectively to flotation after ultrasonic pretreatment, they must persist after ultrasonication and remain available for subsequent bubble-particle interactions.

Surfactants are therefore of particular interest because they can simultaneously modify mineral wettability and regulate gas–liquid interfacial properties. Their adsorption on mineral surfaces can increase hydrophobicity and thereby promote bubble-particle attachment [24], [25], [26], whereas their adsorption at gas–liquid interfaces can reduce surface tension and potentially enhance microbubble persistence. This dual interfacial role is particularly relevant to quartz flotation using dodecylamine (DDA). Quartz is a major gangue mineral in many iron ores and is commonly removed by reverse flotation, in which DDA is widely used as a cationic collector [27]. In addition to rendering quartz surfaces hydrophobic, DDA is strongly surface-active at the gas–liquid interface and can substantially reduce surface tension [24]. It may therefore influence quartz flotation through two coupled pathways: modification of the quartz surface and regulation of *UG* microbubble stability.

Gungoren et al. [28] reported that ultrasonication-induced heating enhances amine activity and improves quartz flotation. Such heating may also reduce gas solubility, thereby increasing gas oversaturation and favoring bubble retention after ultrasonication. Nevertheless, previous studies on ultrasonication in quartz-amine flotation systems have focused primarily on collector adsorption at quartz surfaces [28], [29], [30], whereas the interfacial role of DDA in regulating *UG* microbubbles has received much less attention. In particular, it remains unclear how DDA-induced surface-tension reduction and gas oversaturation jointly regulate the initial post-ultrasonication population and subsequent stability of *UG* microbubbles, and how these persistent microbubbles contribute to quartz flotation.

The post-ultrasonication persistence of both *UG* bulk and surface microbubbles has received limited attention in previous studies. Our previous work [12] established the roles of gas oversaturation and surface wettability in governing the population of persistent *UG* surface microbubbles. Building on this foundation, the present study introduces surfactant adsorption as an additional interfacial factor and investigates its interaction with gas oversaturation in regulating the persistence of both *UG* bulk and surface microbubbles after ultrasonication. Specifically, DDA was employed to elucidate these coupled effects. Unlike our previous study [12], which focused primarily on gas oversaturation and surface wettability, the present work investigates the role of a flotation surfactant in regulating post-ultrasonication microbubble persistence and directly relates this persistence to quartz flotation performance.

The effects of DDA and air oversaturation on the populations of persistent *UG* bulk and surface microbubbles were evaluated using dissolved-air measurements and optical imaging. Silicon wafers were used as model substrates for surface-microbubble observations. Silicon wafers were selected because their native silicon oxide layers exhibit interfacial properties similar to those of quartz. Surface tension and bubble contact angles on silicon wafers in water and DDA solutions were further measured to elucidate the underlying interfacial mechanisms. Finally, the effects of DDA and air oversaturation on quartz flotation following ultrasonication pretreatment were evaluated using bubble pick-up and micro-flotation experiments. This study provides mechanistic insight into the surfactant-enhanced persistence of *UG* microbubbles and offers guidance for their effective application in surfactant-assisted mineral flotation.

## 2 Materials and methods

### 2.1 Materials

In this study, unless otherwise stated, all water used in the following experiments was deionized (DI) water generated at the Helmholtz Institute Freiberg for Resource Technology, and all solutions were prepared with this DI water. Silicon (100) wafers were purchased from Shunsheng GmbH, China. Both hydrophilic (cleaned) and hydrophobic (esterified) silicon wafers were used to investigate *UG* surface microbubbles. Before use, the original wafers were sequentially cleaned with isopropanol, acetone, and DI water in an ultrasonic bath. The cleaned wafers exhibited a water contact angle of approximately 39° [31] and are hereafter denoted as *CS*, with “*C*” representing “cleaned” and “*S*” representing “silicon wafer.” Details of the cleaning procedure are provided in our previous study [31]. Unless otherwise stated, all contact angles reported in this study were measured through the liquid phase. Owing to the presence of a native oxide layer on the silicon wafer, the surface chemistry of the cleaned wafers is comparable to that of quartz. Hydrophobic silicon wafers were prepared by esterifying the cleaned hydrophilic wafers, following the procedure described in Ref. [32]. Briefly, the wafers were reacted with boiling 1-hexanol in a round-bottom flask. The esterified wafers exhibited a contact angle of 80.6° ± 0.7° [21] and are hereafter referred to as *ES*, with “*E*” representing “esterified” and “*S*” representing “silicon wafer.”

Pure quartz particles were used to investigate the role of *UG* surface microbubbles in particle floatability. The quartz sample was supplied by Krantz Mineralienkontor (Germany). The sample was crushed and ground, and the −74 μm particle-size fraction was collected for subsequent experiments. Before use, the quartz particles were cleaned following the procedure described in a previous study [32]. Briefly, the particles were sequentially treated with 0.5 mol/L sulfuric acid, distilled water, 0.5 mol/L sodium hydroxide, and distilled water. During the repeated washing and decantation steps, suspended fine particles were removed with the supernatant. Therefore, no additional desliming step was applied. X-ray fluorescence (XRF) analysis showed that the quartz particles contained approximately 99.27 wt% SiO_2_, confirming their high purity. Further details on the chemical composition and crystalline structure are provided in the Supporting Information (SI).

The cationic surfactant DDA (Sigma-Aldrich, 98 %) was used to modify the surface properties of *CS*, quartz particles, and bubble surfaces.

### 2.2 Solution preparation

In this study, water and DDA solutions were prepared under air-saturated and air-oversaturated conditions, yielding four liquids: air-saturated water, air-oversaturated water, air-saturated DDA solution, and air-oversaturated DDA solution. These liquids are hereafter denoted as *SW*, *OW*, *SDA*, and *ODA*, respectively. In these abbreviations, “*S*” and “*O*” represent “saturated” and “oversaturated”, while “*W*” and “*DA*” represent “water” and “DDA solution”, respectively. The DDA concentration in both *SDA* and *ODA* was 1 × 10^−4^ mol/L. The abbreviations used in this study and their definitions are summarized in Table 1. The DDA solutions were prepared following the procedure described in a previous study [24].

**Table 1:** The abbreviations and their definitions used in this study.

Abbreviation	Definition	Abbreviation	Definition
*UG*	Ultrasonication-generated	*OW*	Air-oversaturated DI water
*CS*	Clean silicon wafer	*SDA*	Air-saturated DDA solution
*ES*	Esterified silicon wafer	*ODA*	Air-oversaturated DDA solution
*SW*	Air-saturated DI water		

The dissolved-air concentration was estimated from the dissolved-oxygen concentration measured with an oxygen sensor (OXYBase, PreSens GmbH, Germany), using the reported molar fraction of oxygen in dissolved air of 35.6 mol%, which is independent of temperature [31].

*SW* and *SDA* were prepared by storing water and DDA solution, respectively, in clean glass bottles with loosely fitted caps under ambient atmospheric conditions (9.85 × 10^4^ Pa and 20 ℃ ± 0.5 ℃) for 72 h to achieve air saturation. The measured dissolved-air concentration was approximately 7.5 × 10^−4^ mol/L in both *SW* and *SDA*, which was slightly lower than the theoretical air-saturation concentration at 20 ℃ (7.7 × 10^−4^ mol/L) [12], [33], likely because of minor fluctuations in temperature and atmospheric pressure during the 72 h equilibration period. Despite this small deviation, *SW* and *SDA* were treated as air-saturated in the subsequent analysis. *OW* and *ODA* were prepared by pressurizing *SW* and *SDA*, respectively, with compressed air until a dissolved-air concentration of 8.8 × 10^−4^ mol/L was reached. Before subsequent tests, the pH of all liquids was adjusted to 10.0 using 1 mol/L NaOH.

### 2.3 Contact angle and surface tension measurements

The liquid-side bubble contact angles on *CS* in *SW* and *SDA* were measured using the captive-bubble method with a contact angle goniometer (OCA50Pro, DataPhysics, Germany) to assess the effect of DDA on wafer wettability. After bubble attachment to the wafer surface in *SDA*, images were recorded for 3 min to determine the time-dependent contact angle. Before bubble attachment in *SDA*, the silicon wafer was immersed for at least 20 min. Increasing the immersion time beyond 20 min did not change the initial bubble contact angle, indicating that DDA adsorption on the silicon wafer surface had approached equilibrium within 20 min.

The maximum bubble pressure method was employed to measure the dynamic surface tension in *SDA* as a function of surface age using a bubble pressure tensiometer (BP100, Krüss). A needle with an inner diameter of 0.45 mm was used to generate the measurement bubbles.

### 2.4 Dissolved-air concentration measurements and bulk microbubble observation

In each experiment, a cubic glass cuvette (50 mm × 50 mm × 50 mm) was filled with 100 mL of the test liquid, which was then ultrasonicated for 2 s. The dissolved-air concentration of each liquid was measured before and after ultrasonication. After ultrasonication, 400 µL of *OW* or *ODA* was transferred with a pipette into a transparent silica liquid cell for bulk microbubble observation under a microscope (Axio Imager.M2m, Zeiss). Images were acquired at 90 s after ultrasonication.

The ultrasonic waves were generated using a horn-type ultrasonic instrument (HD2200, BANDELIN SONOPULS) operating at 20 kHz. The 13 mm-diameter probe was positioned at an immersion depth of 6 mm, with the nominal input power set to 60 W. All ultrasonication treatments in this study were conducted under identical conditions. The ultrasonication conditions of 60 W and 2 s were selected to generate a sufficient number of microbubbles while minimizing thermal effects and maintaining air-oversaturation in *OW* and *ODA* after ultrasonication. The ultrasonication-induced temperature increase was ≤ 0.5 ℃. Accordingly, a post-ultrasonication liquid temperature of 21 ℃ was assumed for all cases in the subsequent analysis.

### 2.5 Surface microbubble observation

Surface microbubbles on the wafer surfaces were imaged from both top and side views during and after the 2 s ultrasonic treatment in each liquid. The experiments were performed in the same cuvette described in Sec. 2.4, containing 100 mL of liquid. Before ultrasonication in DDA solutions, the silicon wafers were immersed for at least 20 min to allow DDA adsorption on the wafer surface to approach equilibrium. Images were acquired using the camera system integrated into the contact angle goniometer. Fig. 1a and b schematically illustrate the observation setups for the top and side views, respectively.

**Fig. 1:**
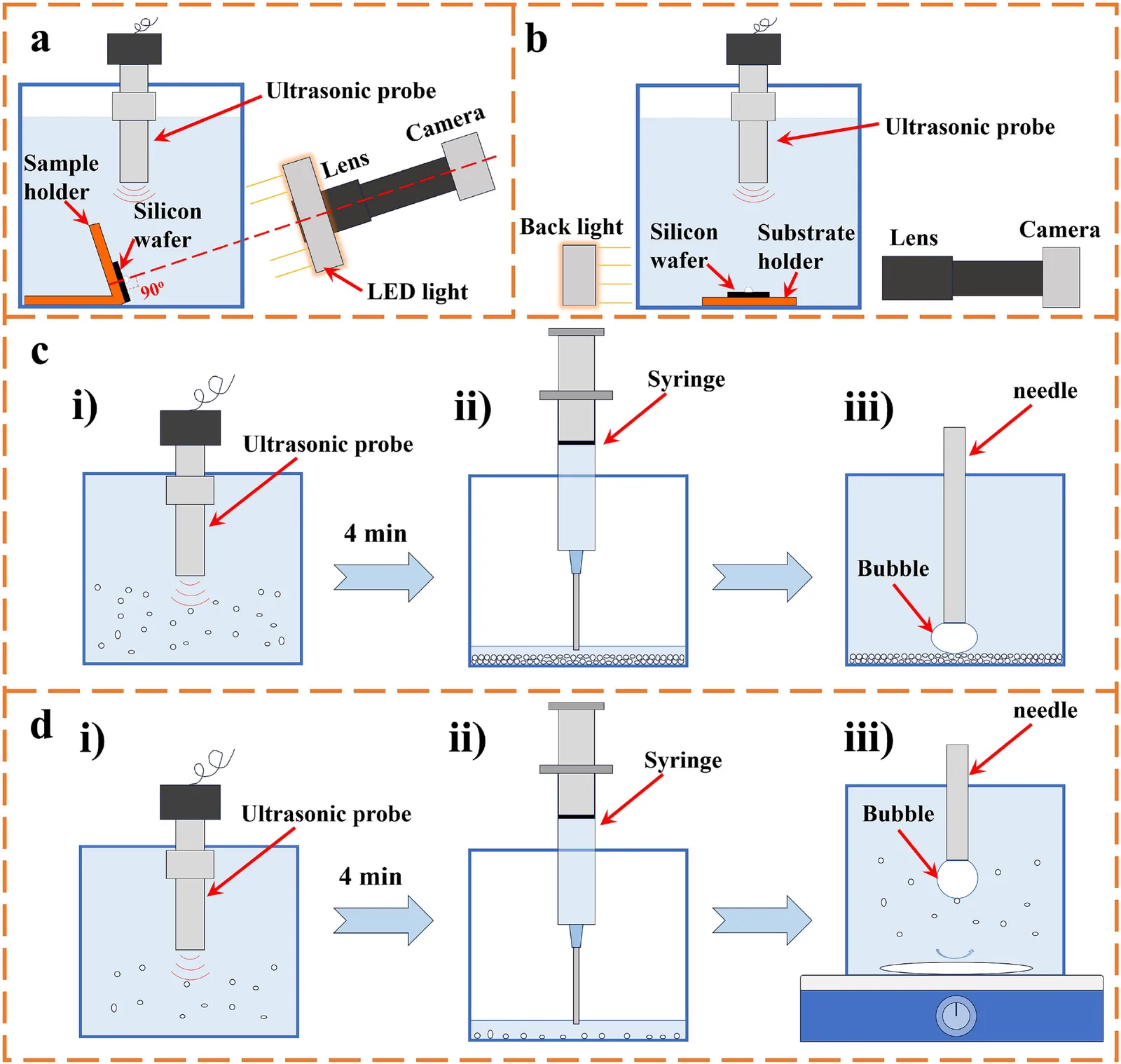
Schematic illustrations of the experimental setups used for (a) top-view and (b) side-view imaging, and the procedure for (c) the single-bubble pick-up and (d) micro-flotation experiments.

For the top-view configuration (Fig. 1a), the camera-lens axis was aligned perpendicular to the wafer surface. The wafer was mounted on a triangular sample holder and illuminated by an LED light strip positioned around the lens. In the top-view images, surface microbubbles were identified as bright spots resulting from reflection at the gas–liquid interface. For the side-view configuration (Fig. 1b), the wafer was mounted on a substrate holder at the bottom of the cuvette and back-illuminated by an LED light source, with the camera-lens axis aligned parallel to the wafer surface. The relative position of the ultrasonic probe with respect to silicon wafer was identical to that used in our previous study [12].

### 2.6 Single-bubble pick-up and micro-flotation experiments

To clarify the role of *UG* surface microbubbles in quartz flotation, single-bubble pick-up tests and micro-flotation experiments were performed using the OCA50 instrument and an XFG flotation cell (Jianfeng, China), respectively. Quartz floatability was evaluated under three conditions: without *UG* surface microbubbles in *SDA*, without *UG* surface microbubbles in *ODA*, and with *UG* surface microbubbles in *ODA*. Experiments in water were not conducted because quartz particles showed negligible attachment to the carrier bubble in the absence of DDA. In both types of experiments, ultrasonication was applied for 2 s, with the ultrasonic probe tip positioned approximately 33 mm above the particle bed.

The bubble pick-up tests were performed according to the procedure schematically illustrated in Fig. 1c [21]: (i) 2 g of quartz particles were sonicated in the cuvette used in Sec. 2.4 containing 100 mL of *SDA* or *ODA* solution. (ii) The cuvette was left undisturbed for 4 min to allow particle settling and clarification of the supernatant, after which 90 mL of the upper liquid was carefully removed using a syringe. The 4 min standing period was selected to ensure that most *UG* surface microbubbles on the particle surfaces in *SDA* had dissolved, whereas those in *ODA* could persist and continue to grow. (iii) The cuvette was then placed on the stage of the OCA50 instrument, and 90 mL of fresh *SDA* or *ODA* was added. An elliptical bubble with a major diameter of 3.1 ± 0.2 mm and a minor diameter of 2.5 ± 0.2 mm was generated at the needle tip using a syringe. The stage was raised at a constant speed until the bubble contacted the settled particle bed at a predefined position and was held in contact for 10 s. After the holding period, the stage was slowly moved downward, and images of the particle-loaded bubble were acquired. For each condition, the bubble pick-up experiment was repeated ten times.

For the micro-flotation experiments (schematically illustrated in Fig. 1d), 2 g of quartz particles were first sonicated in a flotation cell containing 100 mL of *SDA* or *ODA*. The suspension was then left undisturbed for 4 min to allow the particles to settle and the supernatant to clarify, after which 90 mL of the supernatant was carefully removed using a syringe. Subsequently, 90 mL of fresh *SDA* or *ODA* solution was added, and the flotation cell was mounted on the XFG flotation apparatus. The suspension was agitated at 2000 rpm for 1 min before external air was introduced to initiate flotation. Flotation was then conducted for 4 min at the same impeller speed, and the air flow rate was maintained at 30 mL/min. Each experiment was repeated three times under identical conditions.

## 3 Results

### 3.1 Adsorption of DDA on the wafer and bubble surface

DDA can adsorb onto both *CS* and bubble surfaces, thereby increasing the hydrophobicity of *CS* and reducing the gas–liquid interfacial tension. These changes in interfacial properties play important roles in governing the attachment of bulk bubbles to solid surfaces and the stability of microbubbles. The interfacial behavior of DDA is strongly governed by its pH-dependent speciation. Ionic DDA species predominate at pH values below approximately 10.5, whereas the molecular form becomes predominant above this pH [24]. Thus, at pH 10, protonated dodecylammonium ions (RNH_3_^+^) remain the predominant species, while neutral dodecylamine molecules (RNH_2_) are also present. The coexistence of these ionic and molecular species is important for DDA adsorption and organization at both the solid–liquid and gas–liquid interfaces. Notably, previous studies have reported particularly high surface activity of DDA near pH 10, where the surface tension reaches a minimum [24]. Therefore, pH 10 represents a relevant condition for examining the interfacial behavior of DDA in the present system.

The measured bubble contact angle on *CS* in *SW* was 38.7° ± 2.4°. Fig. 2a shows the time-dependent variation in the bubble contact angle on *CS* in *SDA*. Immediately after bubble attachment to the solid surface, the initial bubble contact angle, *θ*, was approximately 73°. It then gradually decreased to approximately 47°. The total time from the onset of bubble formation at the needle tip to bubble attachment on the surface was approximately 1 s. Therefore, the gas–liquid interfacial tension at the beginning of the measurement was expected to be close to that of pure water, approximately 72 mN/m, because the short bubble surface age allowed only limited time for DDA adsorption at the gas–liquid interface. The relatively high initial contact angle confirms that DDA adsorption increased the hydrophobicity of *CS*.

**Fig. 2:**
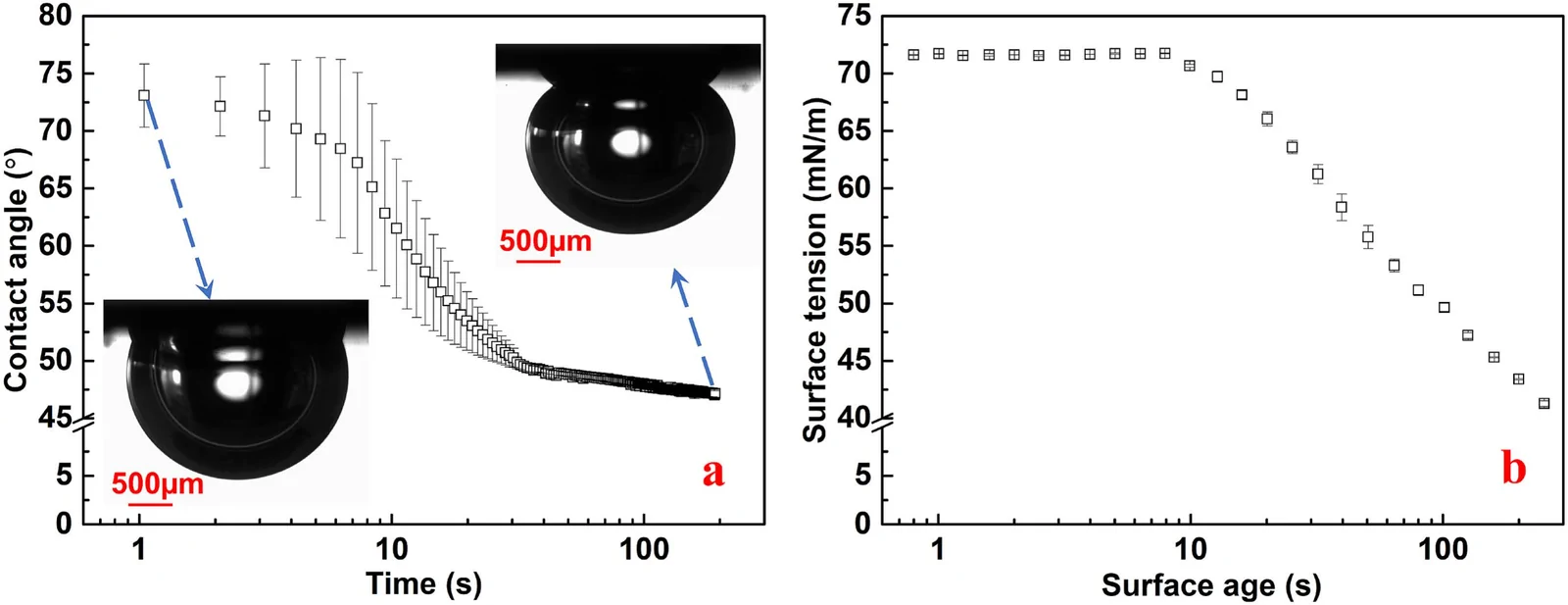
(a) Time-dependent variation in the bubble contact angle in *SDA*; the insets show representative bubble images at the beginning and end of the measurement. (b) Evolution of bubble surface tension in *SDA* as a function of bubble surface age. Error bars represent standard deviations from three measurements conducted under identical conditions.

The subsequent decrease in *θ* may be associated with the progressive adsorption of DDA species at the gas–liquid interface. The contact angle decreased to approximately 62° within about 10 s. To estimate the corresponding change in gas–liquid interfacial tension, an apparent interfacial tension, *σ_GL_*, was estimated using Young’s equation:

(1) $$\begin{array}{c} \sigma_{\mathrm{GL}}\cos\theta =\sigma_{\mathrm{SG}}-\sigma_{\mathrm{SL}} \end{array}$$

where *σ_GL_*, *σ_SG_*, and *σ_SL_* are the gas–liquid, solid–gas, and solid–liquid interfacial tensions, respectively. Assuming that changes in *σ_SL_* and *σ_SG_* were negligible, i.e., that the difference *σ_SG_* - *σ_SL_* remained constant during the measurement, the apparent *σ_GL_* could be estimated from the change in *θ*. Taking *σ_GL_* = 72 mN/m at *θ* = 73°, the apparent *σ_GL_* at *θ* = 62° was estimated to be 44.8 mN/m.

Fig. 2b shows the evolution of the dynamic surface tension as a function of bubble surface age, independently measured using the tensiometer. The surface tension remained nearly constant at approximately 71.6 mN/m for surface ages below 10 s and then gradually decreased to approximately 41.2 mN/m at 250 s, the maximum surface age attainable with the instrument. The observed trend indicates that the interfacial tension had not yet reached equilibrium within the measurement time. Notably, the bubble used in the contact angle measurements, as shown in the insets of Fig. 2a, was much larger than that generated in the dynamic surface-tension measurements, which had a diameter of approximately 0.45 mm. Therefore, the reduction in the apparent gas–liquid interfacial tension during the contact-angle measurements would be expected to occur more slowly than that observed in the dynamic surface-tension measurements. However, the apparent gas–liquid interfacial tension inferred from the contact-angle measurements decreased much more rapidly. This discrepancy suggests possible interfacial redistribution of DDA from the *CS* surface to the bubble interface upon bubble-solid contact, which may locally accelerate the reduction in gas–liquid interfacial tension.

Wang and Miller [34] demonstrated using vibrational sum-frequency spectroscopy (VSFS), that DDA molecules adsorbed at the air–liquid interface can be transferred to the air-quartz interface during film rupture and bubble attachment to a silica surface. Their study was conducted at a low DDA concentration of 1 × 10^−5^ mol/L and a pH of 9.6, under which DDA adsorption at the quartz-solution interface was limited, whereas DDA molecules were enriched at the bubble surface as an ordered monolayer. By analogy, at the higher DDA concentration used in the present study, substantial DDA adsorption may occur on the silicon wafer surface before bubble contact. When freshly generated bubbles, whose interfaces have not yet reached adsorption equilibrium, contact the DDA-covered wafer surface, interfacial redistribution of DDA may occur, potentially involving the transfer of adsorbed DDA from the silicon wafer surface to the bubble surface.

### 3.2 Impact of DDA on UG bulk microbubbles

Ultrasonication can activate gas nuclei in the bulk liquid and generate microbubbles. Meanwhile, the dissolved-air concentration decreases as dissolved air transfers from the liquid phase into the generated bubbles. Fig. 3a shows the dissolved-air concentrations in *SW*, *SDA*, *OW*, and *ODA* before and after ultrasonication. As shown in the figure, the decreases in dissolved-air concentration in *SW* and *SDA* were nearly identical and markedly smaller than those in *OW* and *ODA*. The slight decrease observed in the air-saturated liquids can be attributed to the rapid redissolution of the generated microbubbles before they reached the free liquid surface [12]. This redissolution was driven by the large Laplace pressure of the microbubbles and the undersaturated state of the liquid after ultrasonication. Although DDA adsorption can promote microbubble formation during ultrasonication [5], [6], [7], [8], [9], [10], it can also reduce interfacial mobility and increase interfacial resistance [35], resulting in a lower bubble rising velocity than that in DI water [36]. The slower bubble rise provides more time for bubble dissolution. Consequently, the reduction in dissolved-air concentration in *SDA* was comparable to that in *SW*.

**Fig. 3:**
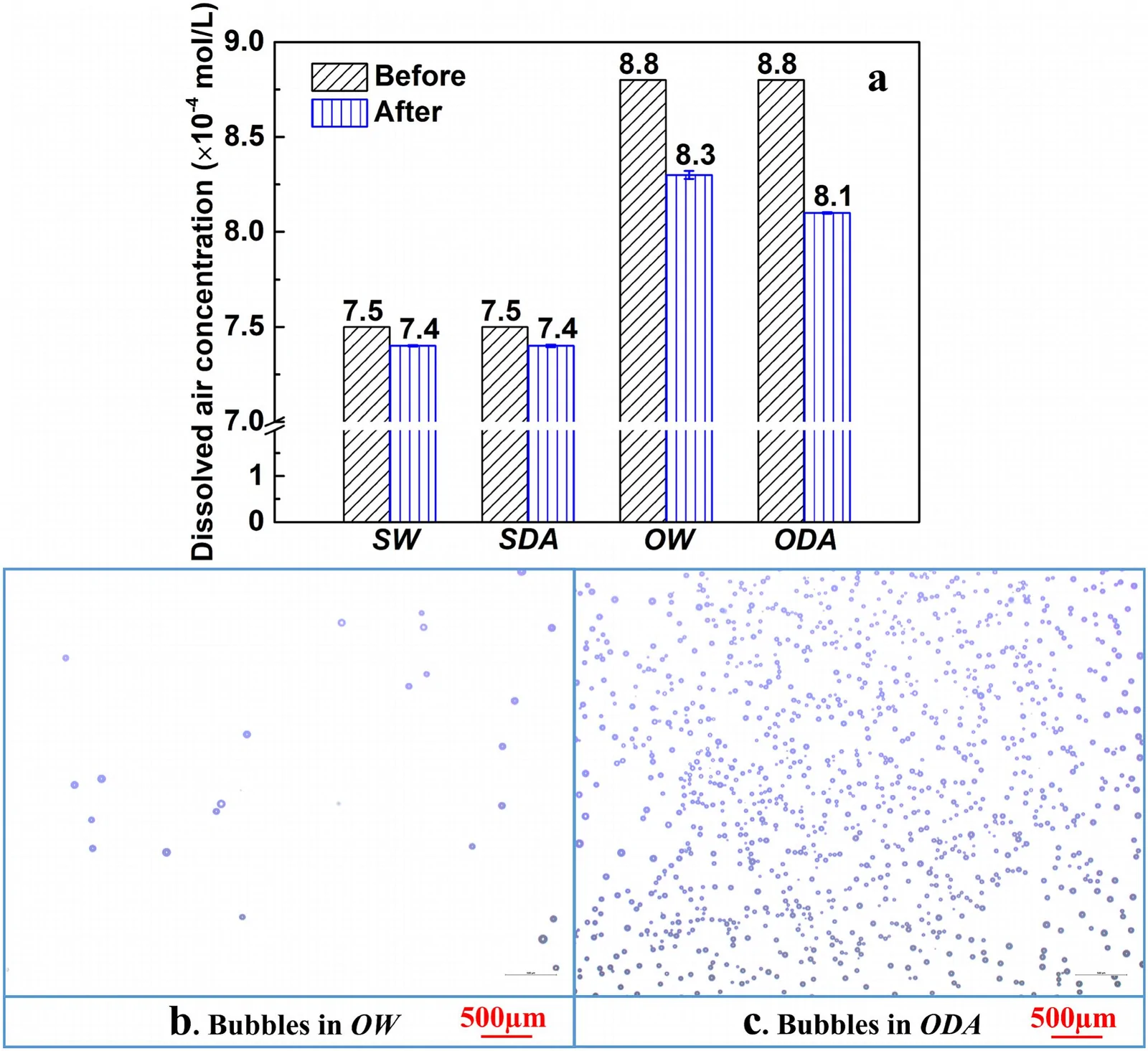
(a) Dissolved-air concentrations in *SW*, *SDA*, *OW*, and *ODA* before and after ultrasonication. Error bars represent standard deviations from three measurements conducted under identical conditions. Representative images of bulk microbubbles in (b) *OW* and (c) *ODA*.

Under air-oversaturated conditions, the generated microbubbles could persist and continue to grow, resulting in much larger decreases in dissolved-air concentration in both *OW* and *ODA*. Notably, the decrease was markedly greater in *ODA* than in *OW*. DDA adsorption at the gas–liquid interface reduces the interfacial tension, which can promote the formation and persistence of a larger microbubble population [5], [6], [7], [8], [9], [10]. More importantly, the reduced interfacial tension lowers the Laplace pressure and thereby allows finer microbubbles to persist and continue growing after ultrasonication. Consequently, more dissolved air was transferred from the liquid phase into the growing bubbles in *ODA*.

Fig. 3b and c show representative images of *UG* bulk microbubbles in *OW* and *ODA* at 90 s after ultrasonication, respectively. Images for *SW* and *SDA* are not shown because most of the microbubbles had rapidly dissolved by 90 s, making it difficult to capture representative bubble images at this time point. At least 10 independent ultrasonication experiments were conducted for each condition, with one image analyzed from each experiment. The number of bubbles in each image was quantified using *ImageJ*. The mean number of bubbles per image was only 9 ± 7 in *OW*, whereas it increased markedly to 891 ± 145 in *ODA*, with the values expressed as mean ± standard deviation. The bubbles in *ODA* were also substantially finer: almost all bubbles in *OW* had diameters greater than 40 µm, whereas a considerable fraction of those in *ODA* were smaller than 20 µm. These results demonstrate that the importance of air oversaturation for the post-ultrasonication persistence of *UG* bulk microbubbles and show that DDA further increases their population and favors the persistence of finer bubbles, consistent with the DDA-induced reduction in gas–liquid interfacial tension.

### 3.3 Impacts of DDA on UG surface microbubbles

Acoustic streaming can transport bulk-nucleated microbubbles toward solid surfaces, where they may attach and form surface microbubbles. Their attachment and subsequent evolution can be influenced by solid-surface wettability, air oversaturation, and gas–liquid interfacial tension. Fig. 4 shows representative top-view images of surface microbubbles observed on *CS* in *OW*, *ES* in *OW*, and *CS* in *ODA* at 0 s (Fig. 4a–c) and 90 s (Fig. 4d–f) after ultrasonication. Each displayed image covers a field of view of 3.1 mm × 2.7 mm and was cropped from the original 5.2 mm × 2.7 mm image to improve microbubble visibility. The images shown in Fig. 4 were contrast-enhanced to facilitate visualization; the original images corresponding to Fig. 4c and 4f are provided in the SI. It should be noted that a small number of fine, low-intensity bright spots may not be retained after image processing and were therefore not included in the quantitative analysis. Corresponding images obtained on *CS* in *SW*, *ES* in *SW*, and *CS* in *SDA* are provided in the SI.

**Fig. 4:**
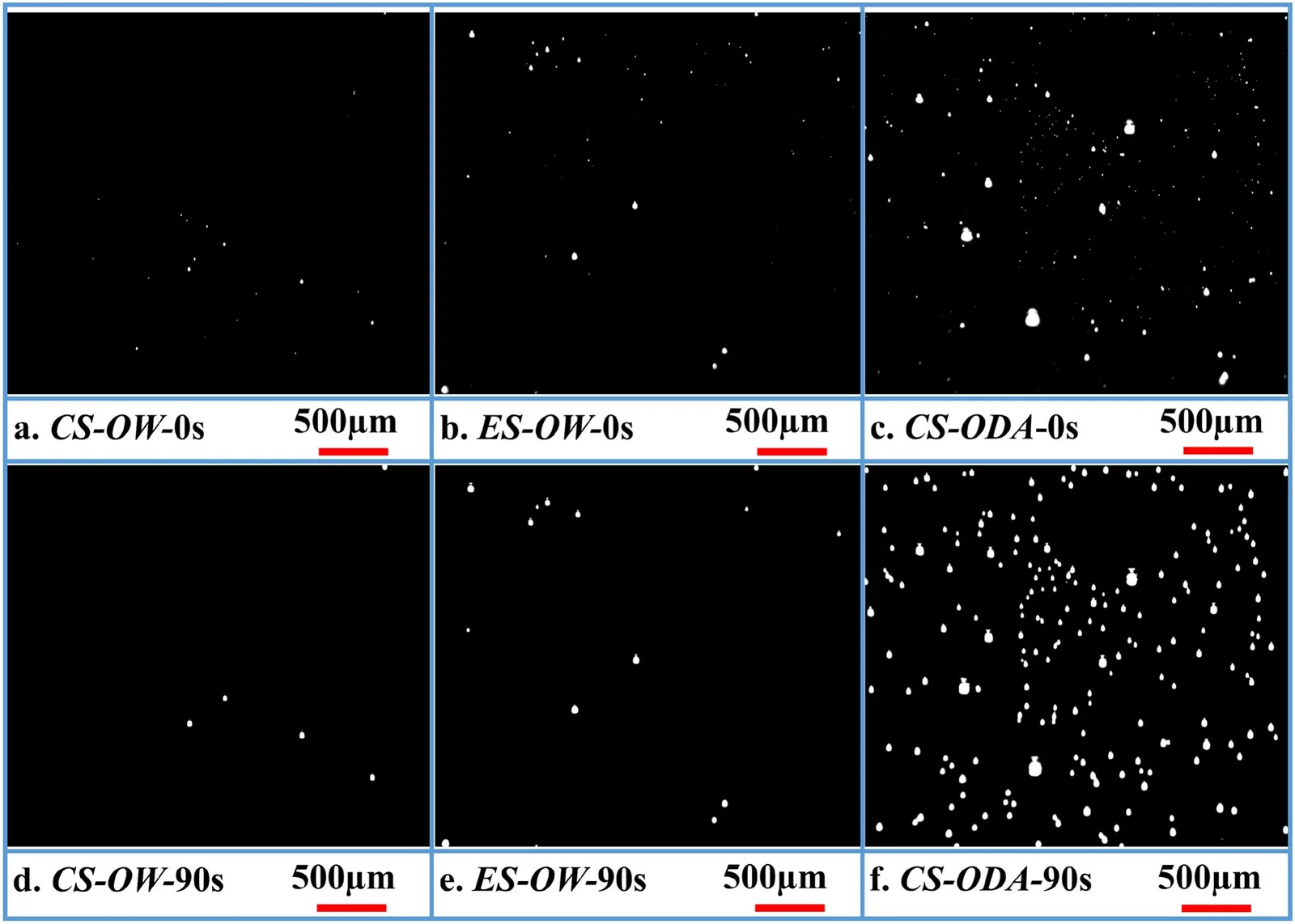
Representative top-view images of surface microbubbles formed on (a, d) *CS* in *OW*, (b, e) *ES* in *OW*, and (c, f) *CS* in *ODA* at (a-c) 0 s and (d-f) 90 s after ultrasonication.

As shown in Fig. 4, finer surface microbubbles tended to dissolve and disappear because of their higher Laplace pressure, whereas relatively larger bubbles persisted and continued to grow. The numbers of surface microbubbles in the contrast-enhanced images at 0 s and 90 s were quantified using MATLAB, and the results are shown in Fig. 5. Under air-saturated conditions, most surface microbubbles disappeared within 90 s, with almost no persistent microbubbles observed in water. In contrast, under air-oversaturated conditions, a substantial fraction of surface microbubbles persisted and continued to grow, demonstrating the important role of air oversaturation in maintaining *UG* surface microbubbles. Surface wettability also strongly affected the surface-microbubble population. In water, both the initial number of bubbles at 0 s and the number remaining at 90 s were markedly higher on hydrophobic *ES* than on hydrophilic *CS*. This result indicates that *UG* microbubbles preferentially attach to hydrophobic surfaces, possibly because the barrier to bubble-surface attachment is lower on such surfaces. These observations are consistent with our previous study [12].

**Fig. 5:**
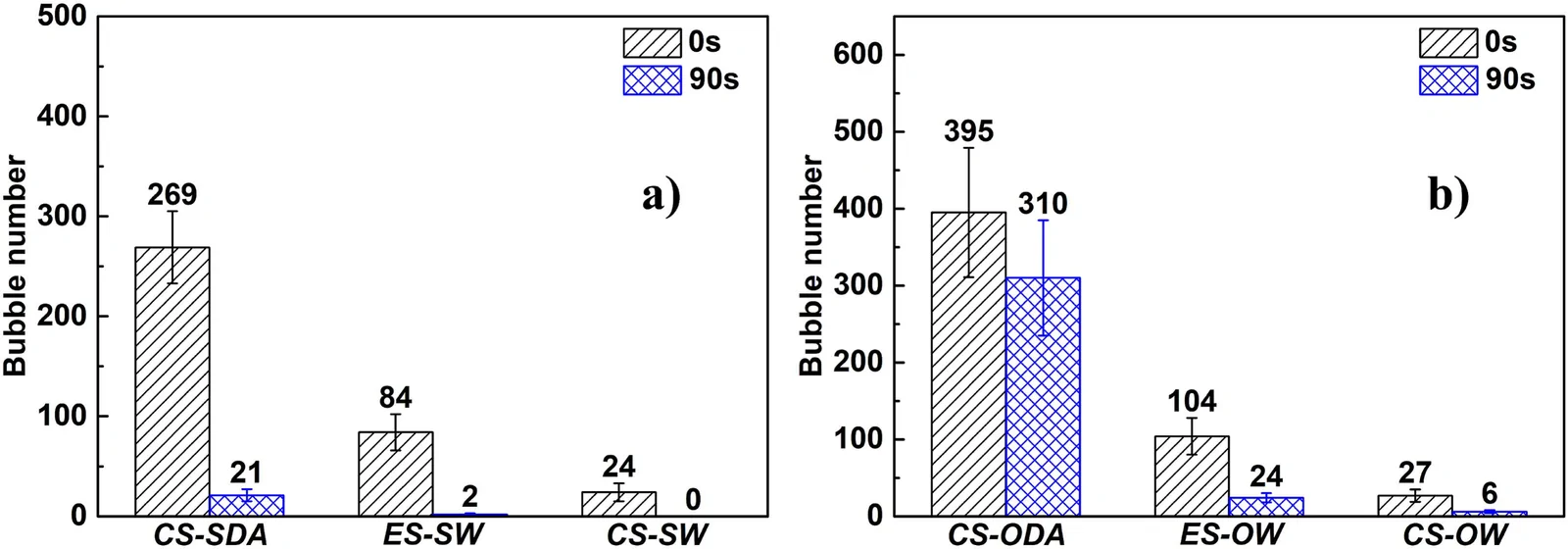
Numbers of surface microbubbles on *CS* and *ES* in (a) air-saturated (*SW* and *SDA*) and (b) air-oversaturated liquids (*OW* and *ODA*) at 0 s and 90 s after ultrasonication. Error bars represent standard deviations from nine experiments conducted under identical conditions.

The initial number of surface microbubbles on *CS* was also markedly higher in DDA solutions than that on *ES* in water, likely reflecting the combined effects of the larger population of *UG* bulk microbubbles available for attachment and the increased hydrophobicity of *CS* following DDA adsorption. In air-oversaturated liquids, the number of surface microbubbles remaining at 90 s depended strongly on the presence of DDA. On *CS* in *ODA*, the number of bubbles remaining at 90 s was approximately 80 % of the initial number at 0 s, whereas the corresponding ratio was only approximately 20 % on *CS* and *ES* in *OW*. As shown in Fig. 4, numerous fine microbubbles remained and continued to grow in *ODA*, whereas bubbles of comparable size were rarely observed at 90 s in *OW*. This difference is consistent with the lower gas–liquid interfacial tension in DDA solutions, which reduces the threshold bubble radius required for diffusive persistence and growth.

To further compare the size ranges associated with bubble persistence, the initial bubble-radius distributions were analyzed using the method established in our previous study [12], in which the bubble diameter was estimated to be approximately 1.4 times the lateral size of the corresponding bright spot. The lateral size of each bright spot was quantified using MATLAB. Fig. 6 shows the cumulative number-based bubble-radius distributions at 0 s on *CS* and *ES* in *ODA* and *OW*, obtained by pooling all bubbles identified across the nine replicate experiments. No substantial differences were observed among the three initial distributions. Here, *r_20_* and *r_80_* denote the bubble radii below which 20 % and 80 % of the total bubble population are found, respectively, in the cumulative number-based radius distribution. Because the number of bubbles remaining at 90 s was approximately 80 % of the initial number on *CS* in *ODA* but only approximately 20 % on *CS* and *ES* in *OW*, *r_20_* for *CS* in *ODA* and *r_80_* for *CS* and *ES* in *OW* can be used as approximate population-level indicators of the size ranges associated with bubble persistence, assuming that bubble loss is dominated by the preferential dissolution of smaller bubbles. The substantially smaller *r_20_* on *CS* in *ODA* than the corresponding *r_80_* values in *OW* therefore suggests that considerably finer microbubbles can persist under air-oversaturated conditions in the presence of DDA. A more direct evaluation of the threshold radius for bubble persistence and growth is presented in Sec. 3.4.

**Fig. 6:**
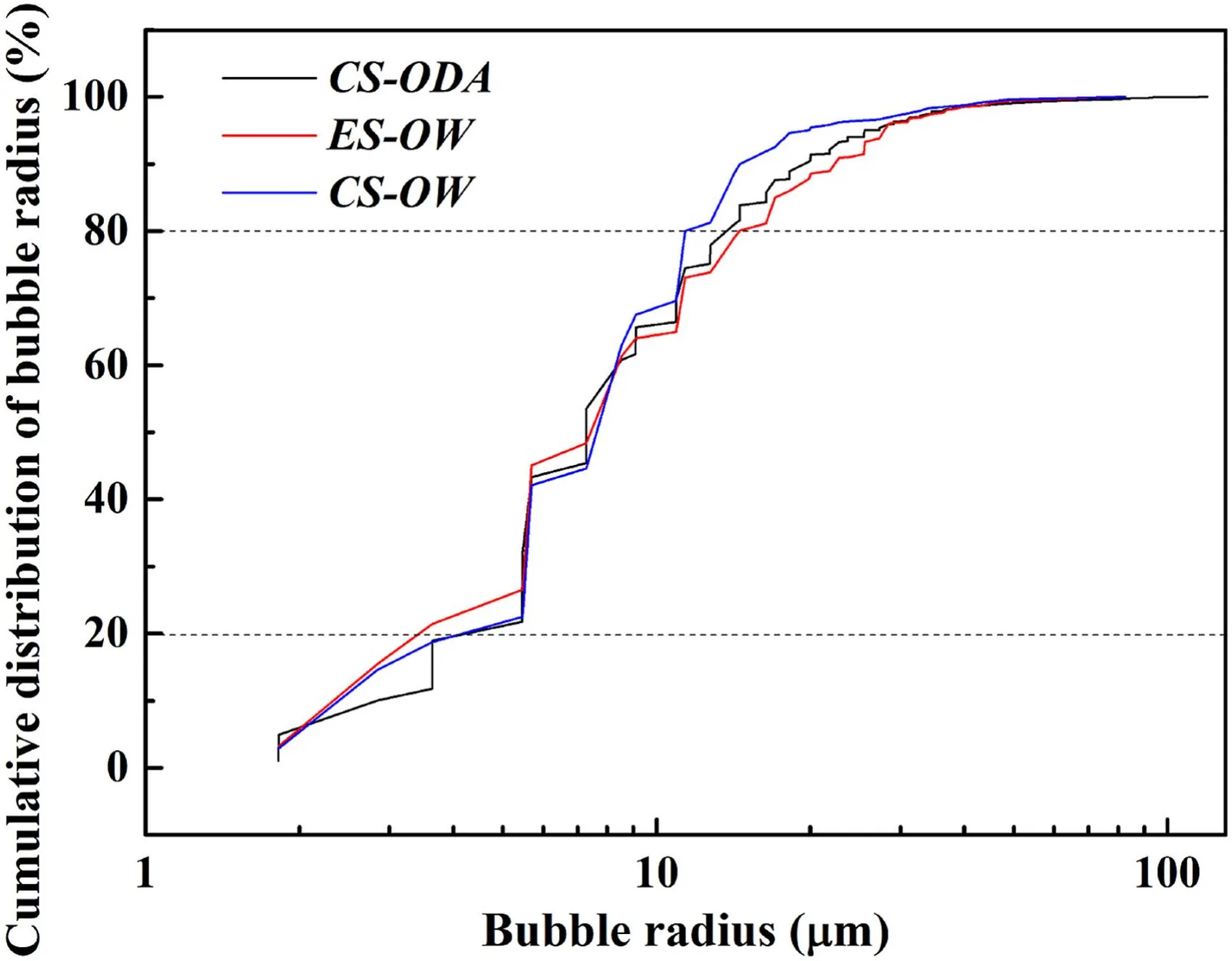
Cumulative bubble-radius distributions at 0 s on *CS* and *ES* in *ODA* and *OW*, obtained by pooling all bubbles identified across nine replicate experiments.

### 3.4 Threshold size for microbubble persistence and growth

This section estimates the threshold radius for bubble persistence and growth by combining Henry’s law with the Laplace pressure. The apparent gas–liquid interfacial tension required for the calculation was estimated from Eq. (1) using the evolution of the microbubble contact angle. If the dissolved-air concentration in the bulk liquid (*c*_∞_) exceeds the dissolved-air concentration at the bubble surface (*c*_s_), dissolved air diffuses into the bubble, leading to bubble growth. Conversely, if *c*_∞_ is lower than *c*_s_, the bubble dissolves and eventually disappears. According to Henry’s law, the dissolved-air concentration at the bubble interface, *c*_s_, is given by Eq. (2) [31]:

(2) $$\begin{array}{c} c_{s}=K^{H,cp}\times \left(P_{0}+\frac{2\sigma_{\mathrm{GL}}}{r_{b}}\right) \end{array}$$

Here, *K^H,cp^*, *P*_0_, *σ_GL_*, and *r_b_* denote Henry’s constant, the atmospheric pressure, the gas–liquid interfacial tension, and the radius of curvature of the bubble interface, respectively. The corresponding air-saturation concentration in the bulk liquid is given by *c_sa,b_* = *K^H,cp^* × *P_0_*. At the transition between bubble dissolution and growth, *c*_s_ = *c*_∞_. Substituting this condition into Eq. (2) yields the threshold radius for bubble persistence and growth, *r*_s_:

(3) $$\begin{array}{c} r_{s}=\frac{2\sigma_{\mathrm{GL}}}{P_{0}\left(\frac{c_{\infty }}{c_{\mathrm{sa},b}}-1\right)} \end{array}$$

Fig. 7 shows images of a microbubble on *CS* in *SDA* at approximately 2 s, 12 s, and 90 s after ultrasonication. The bubble contact angles were 63° at 2 s, 46° at 12 s, and 38° at 90 s. The contact angle of the surface microbubble shown in Fig. 7 decreased more rapidly than that shown in Fig. 2a, suggesting a more rapid decrease in the apparent gas–liquid interfacial tension.

**Fig. 7:**
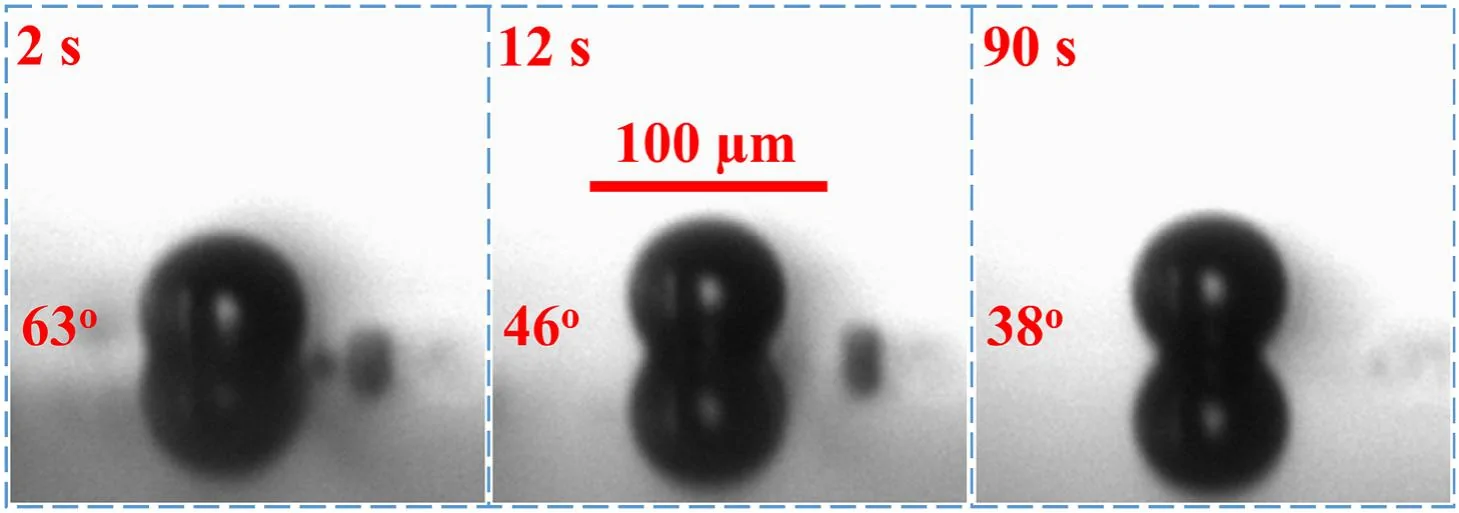
Images of a surface microbubble formed on *CS* in *SDA* at 2 s, 12 s, and 90 s after ultrasonication.

The estimation was based on the same assumptions as those used in Sec. 3.1: namely, that the bubble contact angle was 73° when the gas–liquid interfacial tension was 72 mN/m, and that changes in the solid–liquid interfacial tension, *σ_SL_*, and solid–gas interfacial tension, *σ_SG_*, were negligible. Based on Eq. (1), the apparent gas–liquid interfacial tensions at 12 s and 90 s were estimated to be approximately 30 mN/m and 27 mN/m, respectively. These values are close to the previously reported equilibrium gas–liquid interfacial tension [34]. It should be emphasized that these values were indirectly estimated from the contact-angle evolution under the above assumptions and should therefore be regarded as approximate apparent interfacial tensions rather than directly measured values. The interfacial tension of finer microbubbles is expected to decrease more rapidly because their smaller size can facilitates diffusion-controlled surfactant transport to the gas–liquid interface [37]. Accordingly, for the following analysis, the interfacial tension at the microbubble surface in *ODA* was assumed to reach 27 mN/m before the bubble shrank to the threshold radius.

For the estimation of *r_s_* using Eq. (3), the measured post-ultrasonication dissolved-air concentration was assumed to represent a spatially uniform and temporally constant bulk concentration over the analyzed period, while local gas depletion or enrichment near individual surface microbubbles was neglected. The theoretical air-saturation concentration in both water and DDA solutions at 21 ℃, *c_sa,b_*, was 7.6 × 10^−4^ mol/L [12], [33]. Using *σ_GL_* = 72 mN/m and *c*_∞_ = 8.3 × 10^−4^ mol/L (Fig. 3a) for *OW*, and *σ_GL_* = 27 mN/m and *c*_∞_ = 8.1 × 10^−4^ mol/L (Fig. 3a) for *ODA*, the corresponding threshold radii, *r*_s_, were estimated to be 15.9 µm and 8.3 µm, respectively. The smaller estimated threshold radius in *ODA* is consistent with the observed persistence and growth of finer surface microbubbles under this condition.

For each replicate experiment, the largest initial bubble radius among the bubbles that subsequently dissolved (*r_max_*) and the smallest initial bubble radius among those that subsequently continued to grow (*r_min_*) were identified. The identification procedure is described in the SI. On *CS* in *OW*, the mean *r_max_* and *r_min_* values across the nine replicate experiments were 12.2 µm ± 3.5 µm and 13.5 µm ± 3.4 µm, respectively, with the values expressed as mean ± standard deviation. On *ES* in *OW*, the corresponding values were 12.9 µm ± 2.3 µm and 13.8 µm ± 2.8 µm, respectively. By comparison, on *CS* in *ODA*, both *r_max_* and *r_min_* were substantially smaller, with mean values of 5.2 µm ± 0.8 µm. The overlap between *r_max_* and *r_min_* indicates that bubbles with similar initial radii could either dissolve or continue to grow, suggesting that bubble radius alone did not determine their subsequent fate. Such variations may arise from local heterogeneities in dissolved-air concentration and/or gas–liquid interfacial tension around individual microbubbles, which could not be resolved by the present measurements.

Under the simplified threshold-radius framework, *r_s_*, would nominally be expected to lie between *r_max_* and *r_min_*. The experimentally inferred *r_s_* values for *CS* in both *OW* and *ODA* were smaller than the corresponding theoretical predictions. These discrepancies may likewise arise from local variations in dissolved-air concentration and interfacial tension near individual surface microbubbles, which are not captured by the simplified theoretical model.

### 3.5 Single-bubble pick-up and micro-flotation experiments

Although the greater roughness and heterogeneity of quartz-particle surfaces may affect bubble attachment and the resulting population of persistent surface microbubbles, the general trends identified above are expected to remain applicable to quartz particles. Fig. 8a shows representative images of carrier bubbles after single-bubble pick-up tests with and without *UG* surface microbubbles in *SDA* and *ODA*. The experimental labels used in the following analysis and the corresponding solution conditions are summarized in Table 2.

**Fig. 8:**
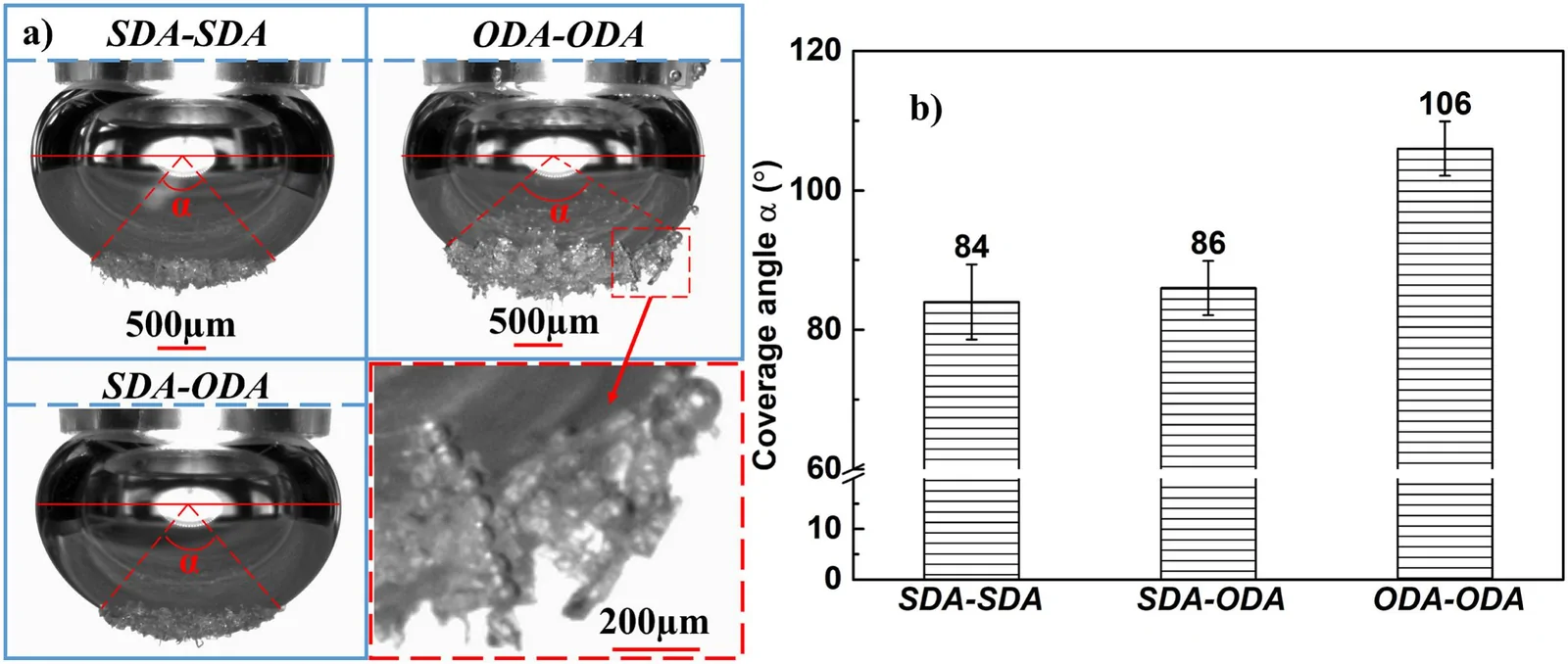
(a) Representative images of particle-loaded carrier bubbles under three conditions: without *UG* surface microbubbles under air-saturated conditions (*SDA*-*SDA*), without *UG* surface microbubbles under air-oversaturated conditions (*SDA*-*ODA*), and with *UG* surface microbubbles under air-oversaturated conditions (*ODA*-*ODA*). In the labels, the first and second terms (*SDA*/*ODA*) denote the solutions applied in steps (i) and (iii) in Fig. 1c, respectively. (b) Particle coverage angles, α, on carrier-bubble surfaces under the corresponding conditions. Error bars represent standard deviations from ten experiments conducted under identical conditions.

**Table 2:** Experimental labels and corresponding solution conditions.

Label	Experimental condition
*SDA*-*SDA*	*SDA* used in both steps (i) and (iii) of the procedures shown in Fig. 1c and d.
*SDA*-*ODA*	*SDA* and *ODA* used in steps (i) and (iii), respectively, of the procedures shown in Fig. 1c and d.
*ODA*-*ODA*	*ODA* used in both steps (i) and (iii) of the procedures shown in Fig. 1c and d.

The use of *ODA* in step (i) yielded a large number of persistent surface microbubbles, whereas few or no persistent surface microbubbles were observed when *SDA* was used. Thus, *SDA*-*SDA*, *SDA*-*ODA*, and *ODA*-*ODA* represent particles without surface microbubbles under air-saturated conditions, particles without surface microbubbles under air-oversaturated conditions, and particles with surface microbubbles under air-oversaturated conditions, respectively. As shown in Fig. 8a, substantially more quartz particles attached to the carrier bubble under the *ODA-ODA* condition. Enlarged images further reveal particle-microbubble agglomerates on the carrier-bubble surface, consistent with a role of surface microbubbles in promoting particle aggregation and attachment through a capillary-bridging mechanism [21].

Fig. 8b compares the particle coverage angle, α, on the carrier-bubble surface under the three conditions. Air oversaturation alone (*SDA-ODA*) did not markedly increase α compared with the air-saturated condition (*SDA-SDA*), indicating that air oversaturation in the absence of pre-existing persistent *UG* surface microbubbles contributed little to particle attachment. In contrast, α increased markedly under the *ODA-ODA* condition, where abundant persistent *UG* surface microbubbles coexisted with air oversaturation. This result indicates that the enhanced particle attachment is primarily associated with the presence of persistent surface microbubbles rather than with air oversaturation alone.

A similar trend was observed in the micro-flotation recoveries (Fig. 9). Air oversaturation alone (*SDA-ODA*) increased quartz recovery by only approximately 3.5 percentage points compared with *SDA-SDA*. In contrast, when abundant persistent *UG* surface microbubbles were present under air-oversaturated conditions (*ODA-ODA*), quartz recovery increased by approximately 12.4 percentage points compared with *SDA-SDA*. The consistency between the single-bubble pick-up and micro-flotation results suggests that persistent *UG* surface microbubbles are a major contributor to the enhanced quartz flotation, primarily by promoting particle attachment to carrier bubbles. These findings further indicate that the combined use of ultrasonication and air oversaturation can substantially enhance DDA-assisted quartz flotation by promoting the persistence of *UG* surface microbubbles.

**Fig. 9:**
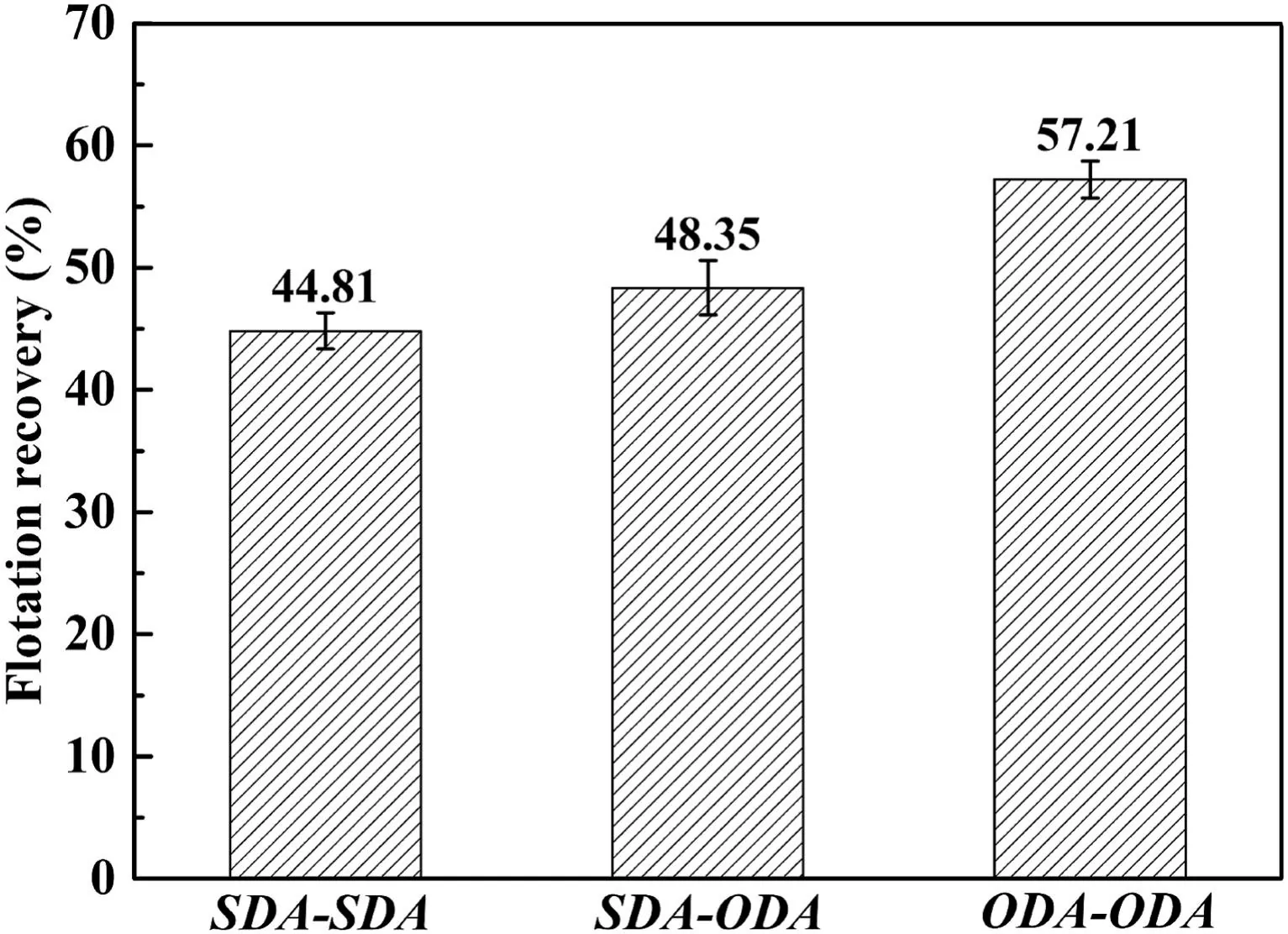
The flotation recoveries of quartz particles under three conditions: without *UG* surface microbubbles under air-saturated conditions (*SDA-SDA*), without *UG* surface microbubbles under air-oversaturated conditions (*SDA-ODA*), and with *UG* surface microbubbles under air-oversaturated conditions (*ODA-ODA*). Error bars represent the standard deviations from three experiments conducted under identical conditions.

## 4 Conclusions

Surfactants can increase the population of microbubbles during ultrasonication; however, after the ultrasonic field is removed, these microbubbles may rapidly dissolve because of their high Laplace pressure. Their post-ultrasonication persistence is therefore critical for subsequent engineering applications but has received comparatively limited attention. In this study, the coupled effects of dodecylamine (DDA), a cationic surfactant widely used as a collector in quartz flotation, and air oversaturation on the population of persistent ultrasonication-generated (*UG*) microbubbles and their contribution to quartz flotation were investigated. The key findings are summarized as follows:

(1) DDA adsorption on silicon wafer surfaces increased surface hydrophobicity. Independent dynamic surface-tension measurements in the DDA solution showed that the surface tension remained nearly constant at approximately 71.6 mN/m for bubble surface ages below 10 s. In contrast, upon bubble-wafer contact in DDA solution, the bubble contact angle decreased much more rapidly in the first 10 s than would be expected from the independently measured surface-tension change. This discrepancy suggests possible interfacial redistribution of DDA from the wafer surface to the bubble interface, which may locally accelerate the reduction in gas–liquid interfacial tension.
(2) The combination of air oversaturation and DDA resulted in a large population of persistent *UG* bulk microbubbles, whereas air oversaturation alone produced only a limited population. Under air-saturated conditions, substantially fewer persistent *UG* bulk microbubbles remained, regardless of the presence of DDA.
(3) DDA increased the initial post-ultrasonication population of *UG* surface microbubbles, likely owing to the larger population of *UG* bulk microbubbles available for surface attachment and the increased hydrophobicity of the silicon wafer following DDA adsorption. However, without air oversaturation, most of these microbubbles subsequently dissolved. Under air-oversaturated conditions, the presence of DDA was associated with a substantially smaller estimated threshold radius for bubble persistence and growth, allowing finer *UG* surface microbubbles to persist. Consequently, a much larger population of persistent *UG* surface microbubbles was observed in air-oversaturated DDA solution than under air oversaturation alone.
(4) The large population of persistent *UG* surface microbubbles in air-oversaturated DDA solution markedly enhanced quartz attachment to carrier bubbles and contributed substantially to the improved flotation recovery, highlighting the potential of combining ultrasonication, air oversaturation, and DDA for quartz flotation.

Overall, this study indicates that the dynamics of surfactant-induced changes in gas–liquid interfacial tension may play an important role in governing the post-ultrasonication persistence of *UG* surface microbubbles. Future studies should investigate how pH and inorganic salt ions affect interfacial-tension dynamics in surfactant solutions and how these effects influence the population and persistence of surface microbubbles, with the aim of further improving surfactant-assisted flotation.

## CRediT authorship contribution statement

**Ming Xu:** Writing – review & editing, Writing – original draft, Validation, Methodology, Investigation, Formal analysis, Data curation, Conceptualization, Resources, Software, Visualization. **Haijun Zhang:** Writing – review & editing, Validation, Supervision, Investigation. **Martin Rudolph:** Writing – review & editing, Supervision, Methodology, Investigation, Formal analysis, Conceptualization, Funding acquisition.

## Declaration of competing interest

The authors declare that they have no known competing financial interests or personal relationships that could have appeared to influence the work reported in this paper.
