# Supplementary material for: Dodecylamine-promoted persistence of ultrasonication-generated microbubbles and its implications for quartz flotation
Source: Ultrason Sonochem. 2026 Oct 26;134:108138. doi: 10.1016/j.ultsonch.2026.108138 (PMC13740880; doi:10.1016/j.ultsonch.2026.108138)
Supplement: Supplementary file 1 [file mmc1.docx]

**Dodecylamine-Promoted Persistence of Ultrasonication-Generated Microbubbles and Its Implications for Quartz Flotation**

1. **Compositions of quartz particles**

The pure quartz particles were analyzed by X-ray diffraction (XRD) and X-ray fluorescence spectrometer (XRF) to determine their crystal structure and chemical composition. The XRD pattern is shown in Fig. S1, and the XRF results are presented in Table S1.

As shown in Fig. S1, quartz was identified as the predominant crystalline phase. The XRF results showed a SiO_2_ content of 99.276 wt.%, confirming the high purity of the quartz sample.

Table S1. Chemical composition of the quartz particles determined by XRF.

| Component | MgO | Al_2_O_3_ | SiO_2_ | K_2_O | CaO | Fe_2_O_3_ |
| --- | --- | --- | --- | --- | --- | --- |
| Content (wt.%) | 0.036 | 0.434 | 99.276 | 0.067 | 0.093 | 0.095 |

Fig. S1. Crystalline phase of the quartz particles determined by XRD.

2. **Original images corresponding to Fig. 4c and 4f**

Fig. S2. Original image corresponding to Fig. 4c.

Fig. S3. Original image corresponding to Fig. 4f.

3. **Top-view images in air-saturated liquids after ultrasonication**

Fig. S4. Representative top-view images of surface microbubbles formed on *CS* in *SW*, *ES* in *SW*, and *CS* in *SDA* at 0 s (upper panel) and 90 s (lower panel) after ultrasonication.

4. **Identification procedure for *r_max_* and *r_min_***

Fig. S5. Representative bubble images acquired at 0 s (upper panel) and 90 s (lower panel).

Fig. S5 shows representative images acquired at 0 s (upper panel) and 90 s (lower panel). During the quantification of the lateral size of each bright spot, individual bubbles in the 0 s image were assigned unique identification numbers. To facilitate manual comparison, the images at 0 and 90 s were each divided into 32 corresponding zones (8 × 4), and corresponding bubbles were identified manually by comparing the two images zone by zone. Bubbles present at 0 s but no longer observed at 90 s were classified as dissolved, whereas bubbles that remained visible and continued to grow were classified as persistent and growing bubbles. Finally, the largest initial radius among the bubbles that dissolved was defined as *r_max_*, while the smallest initial radius among the bubbles that persisted and continued to grow was defined as *r_min_*.
